# Distribution and determinants of the utilization of senior residential care homes in Saudi Arabia: a cross-sectional study

**DOI:** 10.3389/fpubh.2024.1446360

**Published:** 2025-01-07

**Authors:** Saad M. Bindawas, Vishal Vennu, Maha Almarwani, Walid Alkeridy

**Affiliations:** ^1^Department of Rehabilitation Sciences, College of Applied Medical Sciences, King Saud University, Riyadh, Saudi Arabia; ^2^Department of Medicine, College of Medicine, King Saud University, Riyadh, Saudi Arabia; ^3^Department of Medicine, Division of Geriatric Medicine, University of British Columbia, Vancouver, BC, Canada; ^4^General Administration of Home Health Care, Therapeutic Affairs Deputyship, Riyadh, Saudi Arabia

**Keywords:** residential care home, senior, health service, demographic, Saudi Arabia

## Abstract

**Background:**

With the accelerated increase in the population of seniors aged 60 years or older in Saudi Arabia, understanding the utilization of senior residential care homes is crucial for improving service delivery and policy planning to meet the care transformation objectives of Vision 2030.

**Objective:**

To assess the distribution and determinants of senior residential care home utilization across Saudi Arabia’s 13 administrative regions, focusing on predictors of functional dependency among different socio-demographic groups.

**Methods:**

This study analyzed data from 283 Saudi individuals aged ≥65 admitted to social residential care homes in 2021. Variables included age, sex, education level, marital status, region, and reasons for service use. Statistical analyses comprised descriptive statistics, chi-square tests, independent t-tests, and multivariable logistic regression.

**Results:**

The Makkah region had the highest number of senior residential care home users (56.8%; *p* < 0.0001). Most participants were men (67.8%), while women constituted 32.2%. The mean age was 78.9 years (SD = 10.6), with women being significantly older than men (*p* = 0.014). Illiteracy was prevalent (73.5%), particularly among women (82.4% vs. 69.3% for men; *p* = 0.006). Most participants were divorced (68.2%), with higher rates among men (84.9% vs. 33% for women; *p* < 0.0001). The primary reasons for utilizing residential care home services were old age and functional dependency (88.5% of men and 83.4% of women). Multivariable logistic regression indicated that being in the age group 75–84 years (odds ratio [OR] = 1.62, 95% confidence interval [CI] = 1.02–1.81, *p* < 0.001), 85 years and above (OR = 2.63, 95% CI = 1.28–3.11, *p* < 0.001), and being single (OR = 2.43, 95% CI = 1.14–5.13, *p* = 0.019) were significant predictors of old age and functional dependency.

**Conclusion:**

The study highlights regional and socio-demographic variations in senior residential care home service utilization in Saudi Arabia, emphasizing the need for targeted interventions and policies aligned with Saudi Arabia’s Vision 2030 to enhance service accessibility and effectiveness for the aging population.

## 1 Introduction

The global population is aging at an unprecedented rate due to significant social and economic changes. By 2030, one in six people worldwide is projected to be 60 years or older, a figure expected to double to 2.1 billion by 2050, with more than two-thirds residing in low- and middle-income countries (1, 2). In the Arab world, countries such as Algeria, Bahrain, Kuwait, Lebanon, Libya, Morocco, Oman, Qatar, and Tunisia are collectively expected to have 103 million older persons by 2050 (1).

In Saudi Arabia, the aging trend is particularly pronounced. Recent statistics from the General Authority for Statistics indicate that seniors currently constitute approximately 1.3 million individuals, accounting for 5% of the total population (2). Projections suggest that by 2050, this number will surge to over 10 million people aged 60 or older (3).

Three primary factors contribute to the aging population in Saudi Arabia. First, there has been a significant decline in fertility rates over recent years, with United Nations projections indicating a decrease to 1.7 children per woman by 2050 (4). Second, life expectancy has markedly increased—from 42 years in 1950 to 74 years in 2010—and is expected to reach 82 years by 2050, particularly among women (5). Third, declining mortality rates have led to a smaller younger generation and a growing senior population (3). These demographic shifts present substantial challenges in meeting the needs of older adults in Saudi Arabia.

In response to these challenges, the Saudi government has launched numerous initiatives to improve the quality of life for seniors, both in the community and within care homes, under the supervision of the Ministry of Human Resources and Social Development (HRSD). This commitment reflects the nation’s adherence to Islamic values of reverence and care for the older adults. The Ministry operates 12 residential care homes that provide free comprehensive care—including health, social, and psychological support—under Saudi healthcare and social security laws (6). Additionally, the Social Security Agency offers financial and in-kind assistance to seniors and their families, supplying essential items such as wheelchairs, hospital beds, hearing aids, and other necessary devices (7). Innovative practices include the establishment of mobile units to deliver health and care services to older individuals, a strategy also adopted by Bahrain and Oman (1). Other Arab countries have implemented supportive policies, such as tax incentives in Jordan, welfare provisions in Kuwait, long-term care insurance in Egypt, Jordan, and Oman, and pension laws in Lebanon (8).

Beyond financial and material support, the Social Security Agency implements a home care program for seniors through regular follow-up visits within their family environments. As part of the National Transformation Program, the HRSD is initiating the establishment of five model oases tailored to the needs of seniors, offering comprehensive health services, physiotherapy, and recreational facilities. Furthermore, the private sector is permitted to establish 13 specialized civil societies for seniors, ensuring service coverage across all regions of Saudi Arabia (7).

Given the rapid increase in the older adults population globally (9) and in the Arab region (1), particularly in Saudi Arabia (3), it is essential to understand the needs of seniors residing in residential care facilities and the environments in which they live and interact. Addressing these needs is a significant public health concern (10) and is crucial for achieving the care transformation goals outlined in Saudi Arabia’s Vision 2030 (11). Despite the importance of this issue, there has been a lack of comprehensive analysis of the demographics and service utilization patterns in senior residential care homes within Saudi Arabia (12).

Therefore, this study aimed to assess the distribution and determinants of senior residential care home utilization across Saudi Arabia’s 13 administrative regions, focusing on predictors of functional dependency among different sociodemographic groups. By identifying these factors, the study seeks to inform targeted interventions and policies to enhance service accessibility and effectiveness for the aging population, aligning with the objectives of Vision 2030.

## 2 Materials and methods

### 2.1 Study design

This study employed a cross-sectional design to assess the distribution and determinants of senior residential care home service utilization in Saudi Arabia, as recent studies have done in this senior population (13, 14).

### 2.2 Sample size

Given that our study aimed to include all seniors aged 65 years and older residing in government-operated residential care homes across Saudi Arabia, the sample size reflects the total available population meeting the inclusion criteria at the time of data collection. Therefore, a formal sample size calculation was not required.

### 2.3 Study population

The study population included 283 Saudi seniors 65 years and older who were admitted to senior residential care homes in the 13 administrative regions of Saudi Arabia in 2021. The data included both men and women participants and covered various sociodemographic factors.

### 2.4 Ethical consideration

This study did not require formal ethical approval because it utilized publicly available data provided by the Ministry of HRSD. The dataset used in this study can be accessed through the Saudi Open Data Platform.^1^ There was no direct interaction with study participants, and all data were fully anonymized to protect individual identities. The research was conducted following the principles outlined in the Declaration of Helsinki.

### 2.5 Data collection

On March 18, 2021, publicly available data were obtained from the Ministry of HRSD using standardized pre-validated forms to ensure consistent and accurate reporting of variables. These forms capture detailed sociodemographic information, including age, gender, education level, social status, and region of residence. The Ministry of HRSD operates senior residential care homes in Saudi Arabia to offer care and accommodations for all senior men and women citizens unable to take care of themselves and whose family members are also unable to provide the necessary care (9). Government residential care, which encompasses social, medical, and psychological care, as well as a variety of activities such as professional, recreational, and athletic activities, is overseen by a functional body that includes all disciplines through these institutions.

### 2.6 Variables

The primary variables of interest included age, gender, level of education, social status, region and branch, and reasons for the use of services. Age was categorized into 65–74, 75–84, and 85+ years. Gender was recorded as men and women. Education level was classified as illiterate, reads only/reads and writes, elementary or less, middle school, high school, diploma, or university. Marital status was documented as single, married, or divorced. The region and branch referred to the administrative area, such as Makkah or Riyadh, and the specific branch of the senior residential care home. The reasons for service utilization included chronic medical conditions, epilepsy and functional dependency, family breakup/retardation, old age, and functional dependency, psychological conditions, and not reporting.

### 2.7 Data analysis

All statistical analyses were conducted using SAS version 9.4 (SAS Institute Inc., Cary, NC, United States), with a significance threshold set at *p* < 0.05. Descriptive statistics were employed to summarize the data, including means, standard deviations, counts, and percentages. Chi-square tests were utilized to evaluate the significance of categorical variables, while independent *t*-tests were applied for continuous variables. Multivariable logistic regression analyzed the relationship between sociodemographic characteristics and old age and functional dependency. Results were reported as odds ratios (OR) with corresponding 95% confidence intervals (CIs).

In the multivariable logistic regression analysis, the dependent variable was the reason for utilizing senior residential care homes, categorized into two groups: “old age and functional dependency” and “other reasons” (which included chronic medical conditions related to aging, epilepsy, family breakup/retardation, psychological conditions, and unspecified reasons). The “other reasons” category was used as the reference group. Independent variables included gender (with men as the reference group), age groups (65–74 years as the reference group, compared to 75–84 years and 85+ years), educational level (middle school/high school/diploma/university as the reference group, compared to read-only, read & write, unspecified, elementary or less, and illiterate), and marital status (married vs. unmarried).

## 3 Results

**Figure 1:**
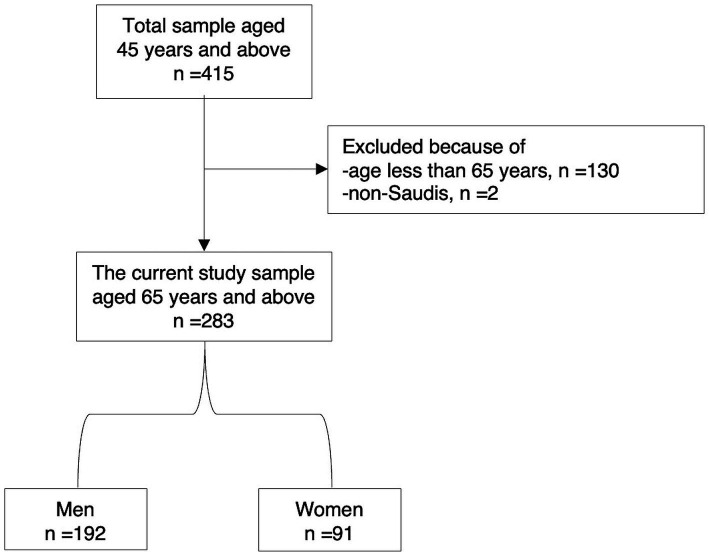
Flow chart of the study participants.

After excluding participants under 65 years of age (*n* = 130) and non-Saudi nationals, a total of 283 Saudi seniors aged 65 years and older were included in the study (Figure 1). Among them, 192 (67.8%) were men and 91 (32.2%) were women. Women were significantly older than men, with mean ages of 80.5 years for women and 77.3 years for men (*p* = 0.014). Age distribution revealed that a higher proportion of men were in the 65–74 years age group compared to women (47.1% vs. 32.2%), whereas a greater proportion of women were aged 85 years and older compared to men (34.5% vs. 21.8%, *p* = 0.037).

Illiteracy was prevalent among the participants, particularly among women (82.4% vs. 69.3% in men, *p* = 0.006). The majority were divorced (68.2%), with a significantly higher proportion among men than women (84.9% vs. 33%, *p* < 0.001). Conversely, a higher proportion of women were married compared to men (27.4% vs. 9.4%). Nearly all participants utilized senior residential care homes (99.6%), with no significant difference between genders (*p* = 0.490).

Geographically, the highest percentage of users was from the Makkah region (56.8%), with men (28.3%) and women (28.6%) equally represented. Riyadh followed with 31.6% of users (15.4% men and 16.2% women), and Al-Madinah accounted for 28.1% of users, with more women (17.6%) than men (10.5%). Other regions with notable usage included Asir (24.2%), Jizan (13.4%), the Eastern Region (12.8%), and Al-Qassim (12.3%), all showing balanced gender distributions. Minimal representation was observed from Tabuk (2.7%), Al-Baha (2.2%), the Northern Border (1.6%), Al-Jawf (1.6%), Najran (1.1%), and Ha’il (0.1%). Branch-specific data indicated that the Makkah branch had the highest percentage of users (43.0%), comprising 23.1% women and 19.9% men. The Abha branch followed with 36.6% of users (20.9% women and 15.7% men), and the Riyadh branch had 36.0% (18.7% women and 17.3% men). Other notable branches included Madinah, Taif, Dammam, and Unizah, each with varying gender distributions (Table 1).

**Table 1:** Demographic characteristics of seniors in senior residential care homes by gender.

Characteristics	Total *n* = 283	Men *n* = 192 (67.8%)	Women *n* = 91 (32.2%)	P^*^
Age (years), Mean (SD)	78.9 (10.6)	77.3 (10.0)	80.5 (11.2)	0.014
Age group, *n* (%) ^#^				0.037
65–74	109 (42.2)	82 (47.1)	27 (32.2)	
74–84	82 (31.8)	54 (31.1)	28 (33.3)	
85 and above	67 (26)	38 (21.8)	29 (34.5)	
Educational level, *n* (%)				0.006
Illiterate	208 (73.5)	133 (69.3)	75 (82.4)	
Read-only/read & write/unspecified	31 (10.9)	20 (10.4)	11 (12.1)	
Elementary school or less	28 (9.9)	27 (14.1)	1 (1.1)	
Middle school/high school/diploma/University	16 (5.7)	12 (6.2)	4 (4.4)	
Marital status, *n* (%)				<0.0001
Married	43 (15.2)	18 (9.4)	25 (27.4)	
Divorced	193 (68.2)	163 (84.9)	30 (33)	
Single	47 (16.6)	11 (5.7)	36 (39.6)	
Service utilized, *n* (%)				0.490
Senior residential care home	282 (99.6)	191 (99.5)	91 (100)	
Other	1 (0.4)	1 (0.5)	-	
Administrative regions, *n* (%)				0.221
Makkah	103 (56.8)	66 (28.3)	37 (28.5)	
Riyadh	85 (31.6)	45 (16.2)	39 (15.4)	
Al-Madinah	45 (28.1)	25 (10.5)	21 (17.6)	
Asir	39 (24.2)	22 (10.0)	17 (14.3)	
Jizan	20 (13.4)	13 (6.8)	7 (6.6)	
Eastern region	35 (12.8)	25 (7.3)	10 (5.5)	
Al-Qassim	36 (12.3)	28 (7.9)	8 (4.4)	
Tabuk	5 (2.7)	3 (1.6)	2 (1.1)	
Al-Baha	5 (2.2)	3 (1.1)	2 (1.1)	
Northern border	2 (1.0)	1 (0.5)	1 (0.5)	
Al-Jawf	6 (1.6)	5 (1.2)	1 (0.4)	
Najran	2 (1.0)	2 (1.0)	0 (0.0)	
Ha’il	7 (1.7)	5 (1.2)	2 (0.4)	
Branch of Senior residential care home, *n* (%)				<0.0001
Makkah	72 (43.0)	47 (23.1)	25 (19.9)	
Abha	58 (36.6)	34 (20.9)	24 (15.7)	
Riyadh	51 (36.0)	0 (0.0)	51 (36.0)	
Madina	66 (33.3)	44 (17.6)	22 (15.7)	
Taif	53 (29.5)	32 (14.1)	21 (14.1)	
Damman	29 (11.2)	21 (6.8)	8 (4.4)	
Unizah	35 (10.5)	35 (10.5)	0 (0.0)	

^*^ Chi-square test. ^**#**^There is missing age data for 25 participants.

**Figure 2:**
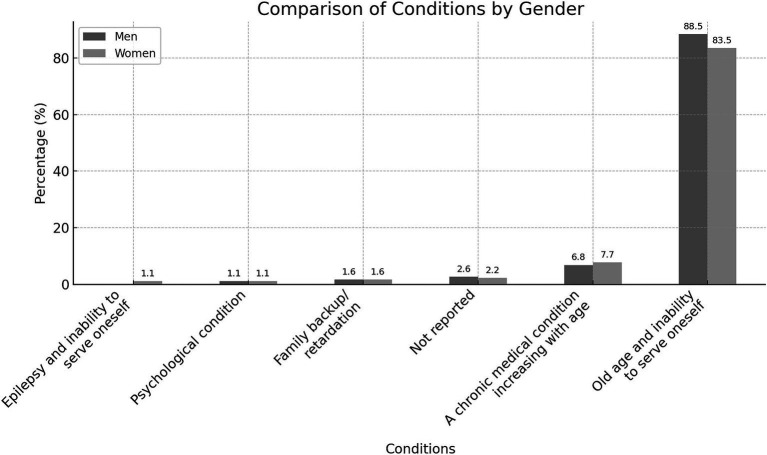
Reasons for utilizing senior residential care home services by gender.

The most common reason for utilizing senior residential care homes was “old age and functional dependency,” reported by 88.5% of participants overall, with similar proportions among men (88.5%) and women (83.5%) (Figure 2). The next most common reason was “chronic medical condition increasing with age,” cited by 14.5% of users (6.8% men and 7.7% women). “Old age and functional dependency” was particularly prevalent among men aged 65–74 years (44.5%), women in the same age group, and those aged 85 years and older (each 35.2%). Among illiterate participants, this reason was reported by 76% of men and 86.8% of women. It was also the primary reason among married men (69.4%) and single women (43.5%), with most single women (66.7%) citing it as their primary reason (Table 2).

**Table 2:** Distribution of social status in men and women who utilized senior residential care home services based on various reasons.

Characteristics	Men			Women		
	Total^**^ *n* = 192 (67.8%)	Old age and functional dependency *n* = 170 (88.5%)	Others* *n* = 22 (11.5%)	Total^**^ *n* = 91 (32.2%)	Old age and functional dependency *n* = 76 (35.2%)	Others* *N* = 15 (16.4%)
Age group, *n* (%)						
65–74	94 (44.5)	80 (43.1)	14 (54.1)	32 (35.2)	26 (34.3)	6 (40)
74–84	53 (28.3)	49 (28.9)	4 (24.3)	27 (29.6)	22 (28.9)	5 (33.3)
85 and above	45 (27.2)	41 (28.0)	4 (21.6)	32 (35.2)	28 (36.8)	4 (26.7)
Educational level, *n* (%)						
Illiterate	184 (69.3)	129 (76)	15 (68.1)	75 (82.5)	66 (86.8)	9 (60)
Read-only/read & write/unspecified	26 (10.4)	24 (14.1)	3 (13.9)	9 (9.8)	5 (6.6)	3 (20)
Elementary school or less	34 (14.1)	9 (5.2)	2 (9.0)	4 (4.4)	3 (3.9)	2 (13.3)
Middle school/high school/diploma/University	20 (6.2)	8 (4.7)	2 (9.0)	3 (3.3)	2 (2.7)	1 (6.7)
Marital status, *n* (%)						
Married	132 (69.7)	118 (69.4)	5 (22.7)	20 (21.9)	13 (17.1)	2 (13.3)
Divorced	18 (9.3)	16 (9.4)	3 (13.6)	29 (31.9)	30 (39.4)	3 (20)
Single	42 (21.0)	36 (21.2)	14 (63.7)	42 (46.2)	33 (43.5)	10 (66.7)

^*^Others, such as a chronic medical condition with increasing age, epilepsy, functional dependency, family breakup/retardation, psychological condition, and not reported. **p < 0.005.

In multivariable logistic regression analysis, being in the age group of 75–84 years (OR = 1.62, 95% CI = 1.02–1.81, *p* < 0.001) and 85 years and above (OR = 2.63, 95% CI = 1.28–3.11, *p* < 0.001) were significant predictors of utilizing senior residential care homes due to “old age and functional dependency.” Additionally, being single was associated with higher odds of using these services for the same reason (OR = 2.43, 95% CI = 1.14–5.13, *p* = 0.019) (Table 3).

**Table 3:** Regression analysis of the association of socio-demographic status with age and functional dependency.

Characteristics	OR (95% CI)	*p*
Gender		
Women (reference)	1.00	
Men	1.02 (0.55—1.89)	0.510
Age group		
65–74 (reference)	1.00	
74–84	1.62 (1.02—1.81)	<0.001
85 and above	2.63 (1.28—3.11)	<0.001
Educational level		
Middle school/high school/diploma/University (reference)	1.00	
Read-only/read & write/unspecified	1.81 (0.41—4.06)	0.433
Elementary school or less	2.23 (0.32—3.22)	0.132
Illiterate	2.42 (0.52—3.27)	0.172
Marital status	3.15 (0.24—4.15)	0.270
Married (reference)	1.00	
Divorced	1.94 (0.85—4.42)	0.113
Single	2.43 (1.14—5.13)	0.019

OR, odds ratio; CI, confidence interval.

## 4 Discussion

This study assessed the distribution and determinants of senior residential care home utilization in Saudi Arabia’s 13 administrative regions, focusing on functional dependency predictors across socio-demographic groups. The study found that senior residential care home utilization in Saudi Arabia’s 13 regions is highest in Makkah, with age groups 74–84, 85 and above, and single individuals being the strongest predictors of old age and functional dependency.

These findings are almost identical to those of an earlier study (15) that described the characteristics of Saudi seniors. According to that research, the average age was 70.1 years, and approximately 70% of seniors lack literacy. A recent randomized controlled pilot study revealed that the average age of Saudi seniors receiving the Makkah senior residential care home is 74.4 years (16). Furthermore, an earlier investigation showed that the average age of senior residents living in nursing homes ranged from 76 to 92 years (17). The current sample had an average age of 78.9 years, and 73.5% of the population, particularly the majority of women (82.4%), were illiterate. A recent study (13) found that health illiteracy was one of the factors preventing some seniors from using senior residential care home services.

An important way to actively address population aging is to appropriately allocate resources to senior residential care homes. However, this study found that Riyadh has the second highest proportion of users of senior residential care homes, after Makkah. A recent Chinese study (18) reported major adverse impacts of aging associated with the unequal distribution of senior residential care homes. Similarly, the results of a recent European study (19) added to the ongoing discussion about the challenges posed by aging populations and their interaction with social care systems.

The results of the study indicate that the main causes of the use of senior residential care home services in Saudi Arabia were old age and functional dependency. However, the best available evidence suggests that motivational interviewing in senior residential care home settings effectively improves participation in physical activity among seniors (20). Forty-six index studies from Australia, Canada, Chile, Hong Kong, the United Kingdom, and the United States provide evidence that interdisciplinary care is used as a structural approach to coordinate care for women in need of multi-agency health and social services (21). Therefore, when designing insurance plans for long-term residential care homes for seniors, policymakers must consider psychological capabilities, psychological motivations, interdisciplinary care (22), and a multidisciplinary approach (23). Furthermore, the establishment of a government agency that could provide equitable assistance to seniors in senior residential care home facilities in Saudi Arabia’s 13 administrative regions (21–23) could be promoted. This agency would use a variety of techniques in the investigation of interventions for senior residential care homes to target vulnerable populations, including the illiterate and those living alone.

Public health priorities continue to include the address of social isolation and loneliness in older adults living alone. The COVID-19 epidemic imposed restrictions that increased the need for services to help these individuals overcome social isolation and loneliness and limited the ability of senior residential care home providers to provide these services. To address loneliness and social isolation in seniors living alone, recent studies (24, 25) that examined the experiences of social care providers suggest that phone calls were the most popular way to promote social interaction, leveraging devices that seniors already had (e.g., smartphones for video calls). Furthermore, a recent study revealed that prioritizing seniors with depressive symptoms in support policies is necessary to encourage aging in place (26).

### 4.1 Implication

Although the quality of healthcare in Saudi Arabia has improved dramatically at all levels over the past few years (27) the senior residential care home service recognizes the need to address social factors to further improve health equity (28). Like many other countries around the world, Saudi Arabia is investing in and trying to improve the quality of social and health care, including the creation of ecosystems of personal data stores in these fields (29). Despite significant improvements, quality barriers persist because the population is growing and the demand for healthcare by patients is increasing. The following categories can be used to group factors that affect the health and quality of senior residential care homes: job satisfaction, workload, culture, literacy, and access to care. Research shows that social determinants affect health, leading to a focus on patient social needs and risk factors (30). This could involve connecting patients with public and private senior residential care home services. However, questions remain about the incorporation of senior residential care homes and infrastructure, even though Saudi Arabia has made notable progress in improving population health in recent decades. The findings of this study will support policy planning initiatives to modernize the country’s old senior residential care home system to achieve the “Vision 2030” goal. This will be achieved by increasing the effective use of these services throughout the country to improve health and reduce health inequities based on specific sociodemographic contexts. “Vision 2030” is the national developmental and economic strategy that Saudi Arabia advocates. The vision outlines the nation’s aspirations to become a global leader by accomplishing three key objectives: a strong economy, an ambitious national agenda, and a lively society. To achieve this, the country introduced in June 2016 a national transformation program (NTP) based on “Vision 2030.” One of the eight topics of the NTP is the transformation of care (12).

### 4.2 Strength and limitations

This study provides a pioneering assessment of the distribution and determinants of senior residential care home utilization across all 13 administrative regions of Saudi Arabia, focusing on key sociodemographic factors such as age, educational level, and marital status. By exclusively utilizing data from centers operated by the Ministry of Human Resources and Social Development, the findings offer a comprehensive and nationally representative overview of public senior care services within the country. The nationwide scope and focus on ministry-affiliated centers enhance the generalizability of the results, providing valuable insights into seniors care practices in the unique cultural and societal context of Saudi Arabia.

However, several limitations should be acknowledged. First, the cross-sectional design of the study limits the ability to establish causal relationships between the identified determinants and the utilization of senior residential care homes. Longitudinal studies are needed to infer causality and understand temporal trends. Second, reliance on data solely from ministry-operated centers may limit the applicability of the findings to the broader spectrum of senior care facilities, excluding private and non-governmental institutions. This focus might affect the generalizability of the results to all forms of seniors care services available in the country. Third, the absence of detailed clinical data constrains the depth of analysis regarding health-related reasons for institutionalization. Categories such as “chronic medical conditions with increasing age,” “epilepsy and functional dependency,” and “psychological condition” were not further specified due to data limitations, hindering a nuanced understanding of specific conditions like neurological disorders, dementia, depression, or anxiety that significantly impact care needs. Lastly, the use of administrative data may introduce biases related to record-keeping practices and does not capture the perspectives or experiences of the seniors themselves.

Future research should aim to include a more diverse range of care facilities, incorporate detailed clinical assessments, and employ longitudinal designs to better elucidate causal pathways. Including data from private and non-governmental centers would provide a more comprehensive picture of senior care utilization across Saudi Arabia. Additionally, qualitative studies capturing individual experiences could enrich understanding and inform targeted strategies for improving the quality, accessibility, and equity of senior residential care services nationwide.

## 5 Conclusion

This study identified the Makkah region as having the highest utilization of senior residential care homes among Saudi Arabia’s 13 administrative regions. Advanced age particularly among individuals aged 75 and above and being married emerged as the strongest predictors of institutionalization due to old age and functional dependency. These findings underscore the critical need for targeted interventions to enhance the accessibility and effectiveness of senior care services for those most at risk. Addressing these needs aligns with the ambitious goals of the Kingdom’s Vision 2030 to improve independence and quality of life for the aging population.

## Data Availability

The raw data supporting the conclusions of this article will be made available by the authors, without undue reservation.

## Footnotes

^1^ https://open.data.gov.sa/
